# Effect of Paeonol on Antioxidant and Immune Regulatory Activity in Hepatocellular Carcinoma Rats

**DOI:** 10.3390/molecules17044672

**Published:** 2012-04-20

**Authors:** Bendong Chen, Mingliang Ning, Guangshun Yang

**Affiliations:** 1 Department of Fifth Hepatic Surgery, Eastern Hepatobiliary Hospital, Second Military Medical University, Shanghai 200438, China; 2 Department of Hepatobiliary Surgery, The General Hospital of Ningxia Medical University, Yinchuan 750004, China; 3 Department of Oncological Surgery, The General Hospital of Ningxia Medical University, Yinchuan 750004, China

**Keywords:** hepatocellular carcinoma, paeonol, oxidative injury, immunity, diethyl-nitrosamine

## Abstract

The study investigated the immunity and antioxidant potential of paeonol by employing a hepatocellular carcinoma (HCC) rat model. Three doses of paeonol (20, 40, 60 mg/kg b.w. orally) were administrated to diethylnitrosamine (DEN)-induced HCC rats. Results showed that paeonol significantly reduced the serum AST, ALT, ALP, GGT, AFU and liver MDA levels, increased serum WBC, TP, ALB, A/G, TNF-α and IFN-γ and liver antioxidant enzymes activities (SOD, CAT, GSH-Px, GR) in HCC rats. Altogether, these results suggest that the paeonol could effectively decrease oxidative injury and improve immunity function in HCC rats.

## 1. Introduction

Hepatocellular carcinoma is the most common and lethal of all cancers. A diversity of dietary [1], endogenous and environmental [2] stimuli mediate hepatocarcinogenesis. Diethylnitrosamine is an *N*-nitroso alkyl compound, categorized as a potent hepatotoxin and hepatocarcinogen in experimental animals, producing reproducible tumors after repeated administration [3,4,5]. The main cause for concern is that diethylnitrosamine is found in a wide variety of foods like cheese, soybean, smoked, salted and dried fish, cured meat and alcoholic beverages [6].

Clinical, epidemiological and experimental studies provide evidence implicating the role of free radicals on the etiology of cancer [7]. Chemical carcinogens acting through free radical metabolites are associated with many biochemical and molecular changes that induces oxidative stress leading to tumor promotion [8]. Nitrosamines compounds induce oxidative stress and retrogradation of the antioxidant defense mechanism due to overproduction of reactive oxygen species (ROS) and lipid peroxidation which result in a wide range of disorders in a variety of rodent organs, especially liver [9]. Drugs with multiple mechanisms of protective and preventive action, including anti-oxidant properties, may be one way forward in minimizing tissue injury in human disease [10]. Cells are also equipped with enzymatic antioxidant mechanisms that play an important role in the elimination of free radicals. Superoxide dismutase (SOD) and catalase (CAT) can counteract the deleterious action of reactive oxygen metabolites and protect from cellular and molecular damage [11]. They also can act as anticarcinogens, and inhibitors at initiation and promotion/transformation stage in carcinogenesis. Reduced glutathione (GSH), glutathione peroxidase (GSH-Px) and glutathione S-transferase (GST) have been assumed as significant markers of chemoprevention owing to their antioxidant and detoxification properties [12].

Paeonol (2'-hydroxy-4'-methoxyacetophenone) is a major phenolic component of Moutan Cortex (*Paeonia suffruticosa*). It has been traditionally used as a Chinese herbal medicine in the treatment of various diseases. It is known to possess anti-inflammatory and anti-proliferative activities. Paeonol was also identified as having various pharmacological and physiological effects such as sedation, hypnosis, antipyresis, analgesia, antioxidation, antibacterial, immunoregulatory, and antitumor properties [13].

As oxidative stress plays a central role in diethylnitrosamine induced hepatotoxicity, the use of antioxidants would offer better protection to counteract liver damage [14]. In the present study, it was decided to evaluate the efficacy of Paeonol, a plant flavanoid, as an antioxidant against diethylnitrosamine induced hepatocellular damage.

## 2. Results and Discussion

Currently, a variety of cytotoxic and antiproliferative agents have been tested in hepatocellular carcinoma (HCC) treatment, which are used alone, or in combination with other drugs or other treatment modalities [15]. Agents with partial response rates near or above 10% include DOX, CDDP and 5-FU [16,17,18]. However, high doses of these drugs lead to severe toxicities, which have a negative effect on patients’ survival. The use of less toxic natural antitumour agents would be desirable [19]. In the clinic, paeonol is usually administered to patients orally or intramuscularly. Paeonol can be quickly absorbed by oral administration. Paeonol has been applied to the clinical therapy of allergic and inflammatory diseases [20], anti-aging, blood pressure control and cardiovascular diseases [21,22], cancer [23], and diabetes mellitus [24].

In the present study, we investigated the antioxidant and immunity activity of paeonol, a natural antitumour and antioxidant agent, in HCC rats. A hepatotoxicity model was successfully produced in Wistar rats by intraperitoneal injection of 200 mg·kg^−1^ diethylnitrosamine (DEN) dissolved in corn oil and then followed by a recovery period of two weeks on food, which was mixed with 2-acetyl-aminofluorene (0.02% AAF) as promoter of hepatocarcinogenesis. As shown in Table 1, there were two dead rats in group II and one dead rat in group III. There were no dead rats in groups I, IV, V and VI.

**Table 1:** Inhibitory effects of tumour growth by paeonol.

Group	Number (n)	Death (n)	Survival rate (%)
I	10	0	100
II	10	2	80
III	10	1	90
IV	10	0	100
V	10	0	100
VI	10	0	100

Values are given as mean ± SD from six rats in each group; group I = Normal group; group II = DEN control group; group III = DEN+ paeonol (20 mg/kg); group IV = DEN+ paeonol (40 mg/kg); group V = DEN+ paeonol (60 mg/kg); group VI = DEN+ Fufang Banmao capsule (15 mg/kg b.w.).

The increase in the activities of plasma AST, ALT, ALP, GGT and AFU indicated that DEN may induce hepatic dysfunction. The enzymes directly associated with the conversion of amino acids to ketoacids are AST and ALT, and are increased in the HCC condition. Table 2 shows the levels of serum AST, ALT, ALP, GGT and AFU were found to be increased in group II rats. Our results were supported by previous studies [25]. Administration of paeonol (20, 40, 60 mg/kg b.w.) caused significant decrease in serum AST, ALT, ALP, GGT and AFU concentrations in group III-V rats when compared with group II rats. Administration of Fufang Banmao capsule (15 mg/kg b.w.) also caused significant decrease in serum AST, ALT, ALP, GGT and AFU concentrations in group III-V rats when compared with group II rats. Paeonol improved liver function by decreasing the serum AST, ALT, ALP, GGT and AFU levels in hepatotoxic rats. Paeonol reduced the ALP as well as AST, ALT, GGT and AFU levels, indicating its protective effect over liver and improvement in its functional efficiency. 

**Table 2:** Effects of paeonol on serum AST, ALP, ALT, GGT and AFU in HCC rats.

Group	AST (U/L)	ALP (U/L)	ALT (U/L)	GGT (U/L)	AFU (U/L)
I	288.82 ± 18.19	141.61 ± 9.62	63.55 ± 3.04	17.57 ± 1.75	30.14 ± 2.77
II	392.16 ± 20.66 ^b^	201.17 ± 12.43 ^b^	91.14 ± 4.01 ^b^	121.63 ± 8.63 ^b^	174.11 ± 9.05 ^b^
III	351.32 ± 19.18 ^d^	180.12 ± 8.92 ^d^	79.14 ± 3.01 ^d^	97.45 ± 5.11 ^d^	152.09 ± 9.29 ^d^
IV	331.24 ± 24.16 ^d^	156.22 ± 6.84 ^d^	70.31 ± 2.81 ^d^	66.43 ± 3.96 ^d^	111.27 ± 7.08 ^d^
V	297.63 ± 15.11 ^d^	126.18 ± 7.84 ^d^	64.92 ± 2.99 ^d^	48.12 ± 2.74 ^d^	81.36 ± 6.83 ^d^
VI	289.68 ± 19.51 ^d^	132.35 ± 9.59 ^d^	60.26 ± 3.85 ^d^	33.71 ± 2.94 ^d^	65.62 ± 5.47 ^d^

^b^ *p* < 0.01, compared with group I; ^c^ *p* < 0.05; ^d^ *p* < 0.01, compared with group II; group I = Normal group; group II = DEN control group; group III = DEN+ paeonol (20 mg/kg); group IV = DEN+ paeonol (40 mg/kg); group V = DEN+ paeonol (60 mg/kg); group VI = DEN+ Fufang Banmao capsule (15 mg/kg b.w.).

White blood cells, or leukocytes, play the main role in immune responses. These cells carry out the many tasks required to protect the body against disease-causing microbes and abnormal cells. Some types of leukocytes patrol the circulation, seeking foreign invaders and diseased, damaged, or dead cells. These white blood cells provide a general—or nonspecific—level of immune protection [26,27]. Serum total protein, also called plasma total protein or total protein, is a biochemical test for measuring the total amount of protein in blood plasma or serum. Serum proteins have many functions, including the transport of other substances, immune defense, blood clotting, and inflammation defense. Serum protein levels are useful for evaluating nutritional status, infection, and various other disorders. Within the human body, albumin is an important component of life [28,29,30]. Albumin in the human body transports essential fatty acids from adipose tissue, otherwise known as fat, to muscle tissue. It is made by the liver. Consequently, decreased albumin levels may be associated with liver disease [31]. An albumin deficiency can lead to medical issues. Albumin/Globulin (A/G) ratio indicates the calculated ratio of levels of these two serum proteins. A low A/G is found in certain liver diseases, kidney disease, myeloma, and inflammation, as well as other disorders [32]. In the present study, DEN has decreased the serum WBC, TP, ALB and A/G. The treatment with paeonol (20, 40, 60 mg/kg b.w.) has significantly enhanced the serum WBC, TP, ALB and A/G in a dose dependant manner (Table 3). This indicated that paeonol could improve immunity function and decrease inflammation in HCC rats. In addition, the treatment with Fufang Banmao capsule (15 mg/kg b.w.) has also significantly enhanced the serum WBC, TP, ALB and A/G (Table 3).

**Table 3:** Effect of paeonol on serum WBC, TP, ALB and A/G.

Group	WBC (×10^9^/L)	TP (g/L)	ALB (g/L)	A/G
I	30.11 ± 1.83	66.39 ± 1.92	38.29 ± 1.72	1.71 ± 0.09
II	22.17 ± 1.08 ^b^	59.18 ± 1.68 ^b^	34.15 ± 1.81 ^b^	1.53 ± 0.11 ^b^
III	25.81 ± 1.22 ^c^	62.71 ± 1.99 ^c^	35.05 ± 1.83	1.55 ± 0.09
IV	27.84 ± 1.65 ^c^	63.92 ± 2.02 ^c^	35.81 ± 1.29	1.64 ± 0.06 ^d^
V	29.62 ± 1.09 ^d^	67.16 ± 2.15 ^d^	37.21 ± 1.47 ^c^	1.69 ± 0.96 ^d^
VI	27.37 ± 1.11 ^d^	70.13 ± 2.74 ^d^	39.84 ± 1.61 ^c^	1.85 ± 0.88 ^d^

^b^ *p* < 0.01, compared with group I; ^c^ *p* < 0.05; ^d^ *p* < 0.01, compared with group II; group I = Normal group; group II = DEN control group; group III = DEN+ paeonol (20 mg/kg); group IV = DEN+ paeonol (40 mg/kg); group V = DEN+ paeonol (60 mg/kg); group VI = DEN+ Fufang Banmao capsule (15 mg/kg b.w.).

TNF-α, a cytokine that plays a role in many inflammatory diseases, is produced by several pro-inflammatory cells (mainly macrophages, but also monocytes, dendritic cells, B-cells, CD^4^^+^ cells, neutrophils, mast cells and eosinophils) and structural cells (fibroblasts, epithelial cells and smooth muscle cells) known to be crucial in the pathogenesis of asthma [33]. Large amounts of TNF-α are generated in response to bacteria or parasitic proteins, but all potentially noxious stimuli ranging from physical, chemical to immunological can rapidly induce production and release of TNF-α. Moreover, TNF-α can also be generated as a consequence of stimulation of a wide range of pro-inflammatory cytokines, including TNF-α itself. TNF-α is also a well-known inducer of the inflammatory response and a regulator of immunity. Its inflammatory properties are classically mediated by means of a wide variety of pro-inflammatory cytokines, including IL (interleukin)-1, IL-2, IL-4, IL-6, IL-10, IL-12, IFN-γ (interferon-γ) and TGF-β (transforming growth factor-β), generated mainly through NF-κB (nuclear factor κB) activation [34,35,36]. In the group II, serum TNF-α level was found to be significantly higher, whereas IFN-γ level was lower comparable to the control values (group I). Administration of paeonol (20, 40, 60 mg/kg b.w.) dose-dependently significantly decreased levels of serum TNF-α and increased serum IFN-γ level compared to model control group (group II) (Table 4). Administration of Fufang Banmao capsule (15 mg/kg b.w.) also significantly decreased levels of serum TNF-α and increased serum IFN-γ level compared to model control group (group II) (Table 4). 

**Table 4:** Effect of paeonol on serum TNF-α and IFN-γ.

Group	TNF-α (pg/mL)	IFN-γ (pg/mL)
I	613.72 ± 28.72	619.48 ± 24.91
II	972.38 ± 52.38 ^b^	328.32 ± 15.51 ^b^
III	904.22 ± 42.57 ^a^	420.24 ± 18.94 ^d^
IV	811.37 ± 38.42 ^d^	512.33 ± 19.66 ^d^
V	677.83 ± 32.64 ^d^	592.83 ± 25.62 ^d^
VI	614.52 ± 42.52 ^d^	631.7 ± 39.32 ^d^

^b^ *p*< 0.01, compared with group I; ^d^ *p*< 0.01, compared with group II; group I = Normal group; group II = DEN control group; group III = DEN+ paeonol (20 mg/kg); group IV = DEN+ paeonol (40 mg/kg); group V = DEN+ paeonol (60 mg/kg); group VI = DEN+ Fufang Banmao capsule (15 mg/kg b.w.).

In the group II, the spleen CD_4_^+^ and ratio of CD_4_^+^ to CD_8_^+^ were found to be significantly decreased, whereas spleen CD_8_^+^ markedly increased compared to the control group (group I). Rats administered with paeonol were found to significantly increase spleen CD_4_^+^ and ratio of CD_4_^+^ to CD_8_^+^, decrease CD_8_^+^, when administered at 20, 40, 60 mg/kg b.w., respectively (Table 5). 

**Table 5:** Effect of paeonol on spleen CD_4_^+^, CD_8_^+^ and CD_4_^+^/CD_8_^+^.

Group	CD_4_^+^ (%)	CD_8_^+^ (%)	CD_4_^+^/CD_8_^+^
I	48.08 ± 4.19	25.86 ± 1.76	1.85 ± 0.24
II	31.47 ± 2.76 ^b^	33.16 ± 1.95 ^b^	0.94 ± 0.39 ^a^
III	39.52 ± 2.72 ^c^	24.76 ± 1.38 ^d^	1.59 ± 0.22 ^c^
IV	40.13 ± 3.82 ^c^	25.15 ± 1.81 ^d^	1.59 ± 0.28 ^c^
V	39.21 ± 4.52 ^c^	24.17 ± 2.83 ^d^	1.62 ± 0.23 ^c^
VI	37.58 ± 4.81 ^c^	22.13 ± 2.21 ^d^	1.68 ± 0.34 ^c^

^a^ *p* < 0.01; ^b^ *p* < 0.01, compared with group I; ^c^ *p* < 0.01; ^d^ *p* < 0.01, compared with group II; group I = Normal group; group II = DEN control group; group III = DEN+ paeonol (20 mg/kg); group IV = DEN+ paeonol (40 mg/kg); group V = DEN+ paeonol (60 mg/kg); group VI = DEN+ Fufang Banmao capsule (15 mg/kg b.w.).

The impaired liver functions may be due to the oxidative tissue damage caused by the massive production of reactive oxygen species (ROS) and disturbance in the protective physiological moieties (as antioxidant defense mechanism systems) causing lipid peroxidation, a process leading to damage to the macromolecules in vital biomembranes [37,38,39]. The anti-oxidative defense system may scavenge ROS that play an important role in the initiation of lipid peroxidation. This defense system operates through enzymatic and nonenzymatic components. GSH-Px and SOD are key enzymes in the body to eliminate free radicals. SOD can change the highly toxic superoxide anions (O_2_^−^) to O_2_ and H_2_O_2_, then H_2_O_2_ and O_2_^−^ react while the ironchelating compounds exist, and produce OH^−^ which has strong activity; meanwhile, GSH-Px can further catalyzed the reduction of GSH and H_2_O_2_, oxidize H_2_O_2_ to H_2_O and prevent the production of highly toxic OH [40]. CAT causes direct breakdown of hydrogen peroxide to oxygen and water [41]. GR is the key enzyme in the conversion of oxidized glutathione (GSSG) back to the reduced form (GSH) [42]. GSH scavenge the electrophilic moieties produced by toxic chemicals and conjugate them to less toxic products [43]. Most of the hepatotoxic chemicals damage liver cells mainly by inducing lipid peroxidation and other oxidative damages in liver. Enhanced lipid peroxidation produced during the liver microsomal metabolism of ethanol may result in hepatitis and cirrhosis [44]. In our present study, Table 6 shows the activity of SOD, CAT, GSH-Px and GR as well as MDA and GSH concentration in liver of all groups of rats. Rats in group II showed a significant decrease in the activity of these enzymes (SOD, CAT, GSH-Px and GR) and the level of GSH and a significant increase in the level of MDA. The results were in agreement with previous studies [45,46]. Rats treated with paeonol showed a significant increase in the activity of SOD, CAT, GSH-Px, GR and the level of GSH as well as a significant decrease in the levels MDA in the liver of the treated rats when compared to group II rats. This indicated that paeonol may alleviate oxidative injury in HCC rats. Likewise, rats treated with Fufang Banmao capsule (15 mg/kg b.w.) showed a significant increase in the activity of SOD, CAT, GSH-Px, GR and the level of GSH as well as a significant decrease in the levels MDA in the liver of the treated rats when compared to group II rats.

**Table 6:** Effect of paeonol on liver MDA, GSH, SOD, CAT, GSH-Px and GR activities.

Group	MDA(nmol/mg prot)	GSH (nmol/mg)	SOD (U/mg prot)	CAT (U/mg prot)	GSH-Px (U/mg prot)	GR (U/mg prot)
I	3.53 ± 0.16	74.39 ± 3.61	321.32 ± 15.05	63.28 ± 2.11	51.81 ± 1.77	37.24 ± 1.42
II	7.84 ± 0.39 ^b^	39.19 ± 1.74 ^b^	161.19 ± 11.39 ^b^	32.14 ± 1.72 ^b^	26.02 ± 1.41 ^b^	16.93 ± 1.39 ^b^
III	5.98 ± 0.22 ^d^	52.77 ± 1.91 ^d^	199.48 ± 11.52 ^d^	44.17 ± 1.93 ^d^	35.63 ± 1.62 ^d^	27.18 ± 1.66 ^d^
IV	4.77 ± 0.17 ^d^	67.14 ± 2.82 ^d^	267.14 ± 11.62 ^d^	58.11 ± 2.72 ^d^	45.28 ± 1.83 ^d^	34.14 ± 1.95 ^d^
V	3.68 ± 0.21 ^d^	75.03 ± 2.92 ^d^	316.13 ± 14.71 ^d^	62.63 ± 2.99 ^d^	52.33 ± 2.41 ^d^	38.05 ± 1.51 ^d^
VI	5.05 ± 0.29 ^d^	69.41 ± 2.77 ^d^	287.48 ± 19.66 ^d^	58.47 ± 2.81 ^d^	48.51 ± 2.73 ^d^	35.62 ± 1.88 ^d^

^b^ *p* < 0.01, compared with group I; ^d^ *p* < 0.01, compared with group II; group I = Normal group; group II = DEN control group; group III = DEN+ paeonol (20 mg/kg); group IV = DEN+ paeonol (40 mg/kg); group V = DEN+ paeonol (60 mg/kg); group VI = DEN+ Fufang Banmao capsule (15 mg/kg b.w.).

The histopathological changes in liver tissues induced by DEN in normal and paeonol-supplemented rats were examined. Livers from control rats revealed normal architectures. Livers from rats treated with DEN alone showed clear signs of severe hepatic injury manifested as areas with periportal and perivascular inflammatory infiltrates with diffuse ballooning degeneration, intra-acinar lymphoplasmacytic and polymorphonuclear infiltrates with adjacent hepatocytes exhibiting feathery degeneration and regenerative cellular changes, proliferation of vascular channels are also noted in several areas, binucleation, acidophilic bodies and nuclear enlargement are some of the regenerative cellular changes noted in most of the sections, granuloma formation accompanied by hepatocytes exhibiting ballooning degeneration and multinucleated giant cells are seen, within some of the granulomas. Livers from rats treated with paeonol plus DEN showed fewer neoplastic cells, near normal architecture, and significant improvement in liver histopathology.

## 3. Experimental

### 3.1. Material

Paeonol was purchased from Xian HaiXuan Biology Science Technology Ltd. Its purity was 99%. Fufang Banmao capsule is a chinese medicine which is used for liver disease.

### 3.2. Experimental Design

Sixty Wistar rats, 240–270 g, were acclimatized under controlled conditions in the laboratory for one week before use. The rats were divided into six groups (I, II, III, IV, V and VI) and each group contained 10 rats. Rats in groups II, III, IV, V and VI were induced with cancer by intraperitoneal injection of 200 mg·kg^−1^ diethylnitrosamine (DEN) dissolved in corn oil, followed by a recovery of two weeks on food, which was mixed with 2-acetylaminofluorene (0.02% AAF) as promoter of hepatocarcinogenesis. Then the rats were left for two weeks. The rats in group I were not induced with cancer but injected once intraperitoneally with corn oil and acted as normal control. Paeonol (20, 40, 60 mg/kg b.w.) was orally given to rats by gavage in groups III, IV and V, respectively during the experiments. Fufang Banmao capsule (15 mg/kg b.w.) (positive control) was orally given to rats by gavage in group VI during the experiments, but rats in group II did not receive any other treatment.

At the termination of experiment, the rats were weighed and complete autopsies were performed after the rats had been sacrificed by decapitation under ether anesthesia. Blood was collected by heart puncture and the serum placed into plain tubes. The samples were centrifuged at 3,800 rpm with a bench centrifuge for 10 min and the serum was stored at −20 °C until the assays were done. Livers were removed and washed in ice-cold 1.15% KCl solution immediately. These samples were processed for biochemistry analysis.

### 3.3. Bichemical Analysis

TNF-α, IFN-γ were measured using commercially available kits. Routine biochemical tests (AST, ALP, ALT, GGT, AFU) were measured using standard methods and matched reagents (Hitachi 7600 Analyzer, Hitachi, Japan; Wako Diagnostics Reagents, Wako Pure Chemical Industries Ltd., city, Japan).

White blood cell count (WBC) was assessed using an automatic multichannel blood cell counter (Sysmex SE-9000, Sysmex Co., Hyogo, Japan). Total protein (TP), and albumin (ALB) were determined by an autoanalyzer (model JCB MB8, Nihon Denshi, Tokyo, Japan). Globulin (Glob) and the Alb/Glob (A/G) ratio were calculated on the basis of TP and ALB concentrations.

Spleens were cut and fixed by immersing in 4% paraformaldehyde immediately. The specimens were embedded in regular paraffin wax and cut into 4-μm-thick sections. Tissue sections were deparaffinized and rehydrated in PBS. Endogenous peroxidase activity was blocked by incubation with 3% H_2_O_2_/PBS for 10 min. After being immersed in 1% BSA/PBS at 37 for 1 h, the sections were incubated with mouse anti-rat anti-CD_4_, CD_8_ mAb at 4 °C overnight. The specimens were incubated with peroxidase-conjugated goat anti-rat secondary antibody for 2 h at room temperature. Finally, the specimens were immersed in DAB for 10 min. Brown staining cells were defined as positive cells and counted in four high microscope view, the percentage of positive cells and CD_4_^+^/CD_8_^+^ ratio were calculated.

Lipid peroxidation was measured spectrophotometrically by the Satoh [47] method. Serum proteins were precipitated by trichloroacetic acid (TCA) and the mixture was heated for 30 min with thioburbituric acid in 2 M sodium sulphate, in a boiling water bath. The resulting chromogen was extracted with *n*-butyl alcohol and the absorbance of the organic phase was determined at a wavelength of 530 nm. The values were expressed in terms of malondialdehyde (MDA) nmol·mL^–1^ using 1,1,3,3-tetraethoxypropane as the standard.

GSH was estimated in the heart homogenate using DTNB by the method of Ellman [48]. The absorbance was read at 412 nm and the results were expressed as μmol of GSH/g of wet tissue.

The activity of tissue superoxide dismutase (SOD) was measured by the Marklund and Marklund method [49]. Superoxide anion is involved in the autooxidation of pyrogallol at alkaline pH 8.5 and is inhibited by SOD, which can be determined as an increase in absorbance per two minutes at 420 mm. The SOD activity was measured as units·mL^–1^ hemolysate. One unit of SOD is defined as the amount of enzyme required to cause 50% inhibition of pyrogllol auto-oxidation.

### 3.4. Catalase (CAT) Measurement

CAT activity was measured based on the ability of the enzyme to break down H_2_O_2_. The method of Aebi was employed in the assay of CAT activity [50]. Briefly, the tissues were homogenised in isotonic buffer (pH 7.4). The homogenate was centrifuged at 1,000 g for 10 min. Twenty μL of 100-fold diluted tissue supernatant was added to 980 μL of the assay mixture containing 900 μL of 10 mmol/L of H_2_O_2_, 50 μL of Tris HCl buffer (pH 8.0) and 30 μL of distilled water. The rate of decomposition of H_2_O_2_ was monitored spectrophotometrically at 240 nm. CAT activity is expressed as k/mg protein, where k is the first order rate constant.

The activity of glutathione peroxidase was determined by the method of Rotruck *et al.* [51] using hydrogen peroxide as substrate in the presence of reduced glutathione. Values are expressed as μmol of GSH utilized/min/mg protein.

Glutathione reductase, which utilizes NADPH to convert oxidized glutathione to the reduced form, was assayed by the method of Staal *et al.* [52]. One unit of enzyme activity has been defined as nmol of NADPH consumed/min/mg protein.

### 3.5. Histological Analysis

Liver sections were prepared, stained with hematoxylin and Eosin (H&E) and changes in histology were recorded.

### 3.6. Statistical Analysis

The data for various biochemical parameters were analysed using ANOVA and the group means were compared by Duncan’s multiple range test. Values were considered statistically significant when *p* < 0.05.

## 4. Conclusions

The present investigation highlights the antioxidant and immunity regulation potential of paeonol in DEN-induced hepatocarcinogenesis animals. Our study confirms that paeonol can effectively decrease oxidative injury and improve immunity function in HCC rats.
